# Prizes of the Massachusetts Medical Society

**Published:** 1858-11

**Authors:** 


*Prizes of the Massachusetts Medical Society.*—The Massachusetts Medical Society is authorized, by a donation from one of its members, to offer the sum of *one hundred dollars* for the best dissertation adjudged worthy of a prize on the following theme, viz: "To what affections of the lungs does bronchitis give origin?"

The above is open to physicians of every country. The latest article on the relations of bronchitis to other diseases of the lungs was written by Dr. W. T. Gardner, of Edinburgh, in 1850; a review of the paper can be found in the British and Foreign Medico-Chirurgical Review for April, 1852. Each dissertation should be designated by a motto, and accompanied by an envelope, superscribed with the motto, and containing the writer's name and address. The sealed packet accompanying the successful dissertation, will be broken and the author's name announced at the annual meeting of the society in May, 1859.

Dissertations for the above prize must be sent (post paid) to the Corresponding Secretary, Dr. Benj. E. Cotting, Roxbury, Mass., on or before April 15th, 1859.—*Charleston Med. Journal.*

